# Designing “Safer Water.” A Virtual Reality Tool for the Safety and the Psychological Well-Being of Citizens Exposed to the Risk of Natural Disasters

**DOI:** 10.3389/fpsyg.2021.674171

**Published:** 2021-06-10

**Authors:** Luciano Gamberini, Alice Bettelli, Giulia Benvegnù, Valeria Orso, Anna Spagnolli, Michele Ferri

**Affiliations:** ^1^Human Inspired Technology Centre, University of Padova, Padova, Italy; ^2^Department of General Psychology, University of Padova, Padova, Italy; ^3^District Basin Authorities of the Eastern Alps, Venezia, Italy

**Keywords:** virtual reality, river floods, emergency, safety, co-design, affinity diagram, brainstorming, serious game

## Abstract

Virtual Reality (VR) is a popular technology to recreate reality-like scenarios, including dangerous ones, in a realistic but safe way. Because of this potential, VR based research has been applied in psychology studies to provide training and education about how to behave in emergencies such as fires, earthquakes, floods, or typhoons. All these different virtual scenarios have been built to observe how people react to emergencies, what behaviors they adopted, what level of stress is generated, and finally, how to increase citizens' safety. However, there is still little research that shows how Virtual Environment (VE) should be designed to convey appropriate social and psychological “cues” to participants. In this work, we present the result of a series of co-design sessions aiming to bring experts to collaborate in setting up virtual scenarios to increase the quality of life, safety perception, and risk awareness in people living in the proximity of a river. Floods are one of the most threatening climate events, and because of climate change, they are expected to become even more frequent. These disasters have a devastating impact on communities, increasing anxiety and stress levels in citizens living close to rivers. We involved relevant stakeholders to design “Safer Water,” an immersive, interactive, virtual experience to support citizens in psychologically and behaviorally managing pre and post riverbank breakdown situations. HCI experts, hydrogeological and hydraulic engineers, psychologists, and VEs designers took part in affinity diagram and brainstorming activities. Results show how the adopted method was able to generate suitable virtual scenarios, to highlight and classify relevant design requirements, and to find strategies that could improve the quality of life and psychological well-being in “risk-exposed citizens.” The discussion includes a set of open-access guidelines derived from the co-design activities, to support the design of VE for the purposes discussed in the paper.

## Introduction

In recent years, Virtual Reality (VR) has been widely employed to safely and realistically recreate situations that are difficult to investigate in the real world, such as risk environments and emergency contexts. On the one hand, the fact that people tend to respond to situations presented in VR as if they were real (Rovira, 2009) has made VR the perfect tool for studying how people naturally behave to emergencies and which is the level of stress that these situations generate (Gamberini et al., 2003; Ronchi et al., 2015). On the other hand, the fact that virtual simulations highly engage users and allow them to experience firsthand the consequences of their actions has meant that this technology has been effectively applied for educational purposes (often in the form of *immersive serious game*s), for example in teaching the correct procedures to be implemented in risky context (Chittaro and Buttussi, 2015; Çakiroglu and Gökoglu, 2019). Numerous studies have been conducted on different types of emergencies, including those caused by fires (Kinateder et al., 2014; Benvegnù et al., 2019), earthquakes (Tarnanas and Manos, 2001), nuclear risks (Hagita et al., 2020), and military or terroristic attacks (Shendarkar et al., 2008) and, more recently, by climate change, particularly typhoons (Ke et al., 2019) and floods (Fujimi and Fujimura, 2020).

However, whether the purpose of these works is to observe how people react to emergencies or to teach how to deal with them, there is still little research that shows how VEs should be designed to convey appropriate social and psychological cues to participants. In particular, it is not easy to find a balance between the need to represent the emergency realistically, allowing users to be emotionally activated, and the need to clearly suggest the individual and social behavior that people should exhibit in the specific situation represented. In addition, there is a certain disparity in the study of the different risk contexts, at least from a psychological point of view. For example, situations such as fire emergency have been extensively investigated in VR, focusing on evacuation procedures (Kinateder et al., 2014), and training (Williams-Bell et al., 2015; Çakiroglu and Gökoglu, 2019), while for other kind of emergencies, such as river floods, these aspects are still relatively less considered. In particular, river floods are one of the most frequent and threatening climate events, and because of climate change, they are expected to become even more frequent and intense (WHO^1^ River floods have an increasingly devastating impact on communities and territories, diminishing the safety and quality of life of people living in the proximity of a river (Mason et al., 2010).

Very few works on this topic have used VR to study flood-related behaviors. Among these, a recent study (Fujimi and Fujimura, 2020) used immersive VR to test the effectiveness of interventions to encourage evacuation decisions from flash floods. Results showed that the participants reacted to the environmental and social cues provided and that the efficacy of flood evacuation interventions can be empirically examined using VR simulations (Fujimi and Fujimura, 2020). In the work of Zaalberg and Midden (2013), participants assisted to a virtual simulation of a levee breach on a desktop screen, showing (in a post-simulation assessment) an increased motivation to evacuate, seek information, and a stated preference to buy flood insurance compared to the other methods tested. Aside from these and a few other exceptions (Sermet and Demir, 2019), most studies on this topic have focused on how to build VEs that allow non-experts to visualize numerical simulations of changes in hydrological information (Lai et al., 2011; Leskens et al., 2017; Liu et al., 2018; Macchione et al., 2019). In some cases, these 3D environments were employed for flood risk communication and used in public hearings, festivals, and workshops to raise awareness about the risks associated with such extreme events (Lai et al., 2011; Skinner, 2020). However, most of these studies have a common focus on the “technical” construction of the scenario (e.g., development of the numerical model, methods to improve the visual quality, or final rendering of the scenario), while more in-depth research on which contents should be included and how to present them properly is still lacking. Furthermore, as far as we know, none of the previous works have used co-design methodologies to create a VR simulation to improve the quality of life of citizens living near a river by providing them with concrete information to better face the flood emergency.

To fill this gap, in the present work, we describe the result of a series of co-design sessions aiming to bring experts from different fields to collaborate in setting up immersive and interactive virtual scenarios. Indeed, co-design has been effectively applied in other areas related to VR experience. It was mainly used in mental health domain, to create virtual scenarios to facilitate psychological, cognitive, and behavioral interventions for dementia, anxiety disorder, eating disorders, pain management (Tabbaa et al., 2020), and fear of public speaking (Flobak et al., 2019). Co-design techniques have also been effectively applied to develop VR simulations and serious games to promote physical activity (Boger et al., 2018; Eisapour et al., 2020) and to enrich user experience in cultural and naturalistic site (Bettelli et al., 2019). Although co-design does not seem to have been applied in the field of virtual simulation of emergencies, it was still used to design virtual training to manage stressful situations, such as training for police forces in the field of close protection (Lukosch et al., 2012), reentry training for incarcerated women (Teng et al., 2019), and alcohol resistance training for adolescents (Lyk et al., 2020). From a methodological point of view, these works are very heterogeneous in both the co-design techniques used and the type of stakeholders who participated in the activities. Regarding the first point, several techniques were applied in the initial stages of requirements collection and/or experience planning, including affinity diagrams (Bettelli et al., 2019), brainstorming (Lukosch et al., 2012; Lyk et al., 2020; Tabbaa et al., 2020), focus group sessions (Boger et al., 2018; Eisapour et al., 2020), and interviews (Lukosch et al., 2012; Teng et al., 2019). Regarding the second point, these activities mainly involved end users (Lukosch et al., 2012; Teng et al., 2019) and/or experts in the specific application domain, for example kinesiologists for applications to promote physical activity (Eisapour et al., 2020) or managers of private security companies to design virtual training for police forces (Lukosch et al., 2012), while only few studies also involved experts in new technologies and HCI (Bettelli et al., 2019; Tabbaa et al., 2020). On the one hand, the co-design activity helped to obtain valuable feedback from experts (Eisapour et al., 2020), and to identify design recommendations for the specific application domain (Boger et al., 2018; Eisapour et al., 2020). On the other hand, it is not always easy to mediate between the different interests of the stakeholders, possibly slowing down the design process (Lukosch et al., 2012). Aside from the differences in the techniques used, the parties involved and the specific field of application, in these works the adoption of co-design methodologies has allowed the creation of detailed and tailor-made virtual experiences. On this basis, we expect that a co-design approach can be successfully applied to the emergency context, greatly improving the creation process of a river flood scenario.

The general objective was to collect information useful for defining the VE contents and their translation into the virtual educational experience “Safer Water.” Particular attention was paid to the spatial and temporal domain specification, focusing on events, effects, and possible behavioral responses in a situation where the embankment of a river was broken. The following sections will describe the methodologies used, the parties involved in the various activities, and the main results.

## Materials and Methods

To create an immersive and interactive virtual experience that can increase the safety perception and risk awareness in people living in the proximity of a river, a co-design approach was adopted. By doing this, different experts (*N* = 11; Table 1) in several fields were involved in the design process and informed the requirements of the experience. They were experts in hydrology, hydraulics, psychology, and Human–Computer Interaction, and design and implementation of VE (Table 2 describes expertise and previous experience with simulations and VR). These stakeholders took part specifically in the affinity diagram and brainstorming activities.

**Table 1:** Description of the stakeholders involved in the co-design activities: field, expertise, and previous experience in simulation and VR.

**ID**	**Background**	**Stakeholders' professional expertise**	**Previous experience in simulation and VR**
P1 P2 P3	Hydrology	1) Hydraulic and hydrological modeling; 2) Flood risk assessment and management 3) Implementation of flood forecasting systems; 4) Management of forecasting and warning systems with the civil protection; 5) Citizen observatories (especially P1; P2).	No familiarity with virtual reality, but experience in the field of simulations: 1) Implementation of flood simulation models; 2) Simulations of flood events and exercises for the evaluation of technologies and methodologies developed in the context of European Projects; 3) Organization and planning of various civil protection exercises.
P4 P5 P6	Hydraulics		
P7 P8 P9	Psychology and human-computer interaction	1) Interaction design, ergonomics; 2) Participatory design activities and users' study; 3) Creation of storyboards for VR experiences; 4) Testing and evaluation of VR and other new technologies; 5) Persuasive technology.	Use of VR simulations for scientific research purpose in different contexts (e.g., risk management and emergency situations, training and safety in the workplace, clinical area, naturalistic, and cultural heritage, architectural, retail, and product sales).
P10 P11	Design and implementation of VE	1) Creation of storyboards for VR experiences; 2) Architecture design of software in VR; 3) Creation of navigable 3D models optimized for VR and implementation of interactions.	

**Table 2:** Description of the stakeholders' background: age, gender, instruction, and professional experience.

**ID**	**Age**	**Gender**	**Education**	**Professional experience (years)**
P1	30	F	Master's degree in Engineer for the environment and the territory	17
P2	41	F	Ph.D. in Civil and environmental engineering sciences	5
P3	49	M	Ph.D. in Hydrodynamics and environmental modeling	11
P4	42	M	Ph.D. in Environmental hydronomy	6
P5	29	M	Master's degree in Civil engineering, hydraulic specialization	15
P6	35	M	Ph.D. in Hydraulic risk management	20
P7	54	M	Ph.D. in Experimental psychology	30
P8	29	F	Ph.D. student in Neuroscience, technology, and society; master's degree in clinical psychology	4
P9	30	F	Ph.D. student in Neuroscience, technology, and society; master's degree in neuroscience and neuropsychological rehabilitation	5
P10	32	F	Master's degree in Architecture	4
P11	36	M	Not graduated	8

### Gathering the Requirements: Affinity Diagram Sessions

Two affinity diagram sessions were organized, considering two different moments relating to the spatial and temporal domain of the emergency situation. In particular, the goal of the first session was to collect information on the situation prior to the breakdown of a river embankment and on the possible danger indicators that signal with high probability an imminent flood. In comparison, the goal of the second session was to collect information on the emergency created by the rupture of the riverbank and on the consequent behaviors adopted by people. The affinity diagram methodology is widely used to generate, make sense, and organize large amounts of unstructured, far-reaching, and apparently dissimilar qualitative data (Hartson and Pyla, 2012; Lucero, 2015).

#### First Session: Before the Embankment Breakdown

**Figure 1:**
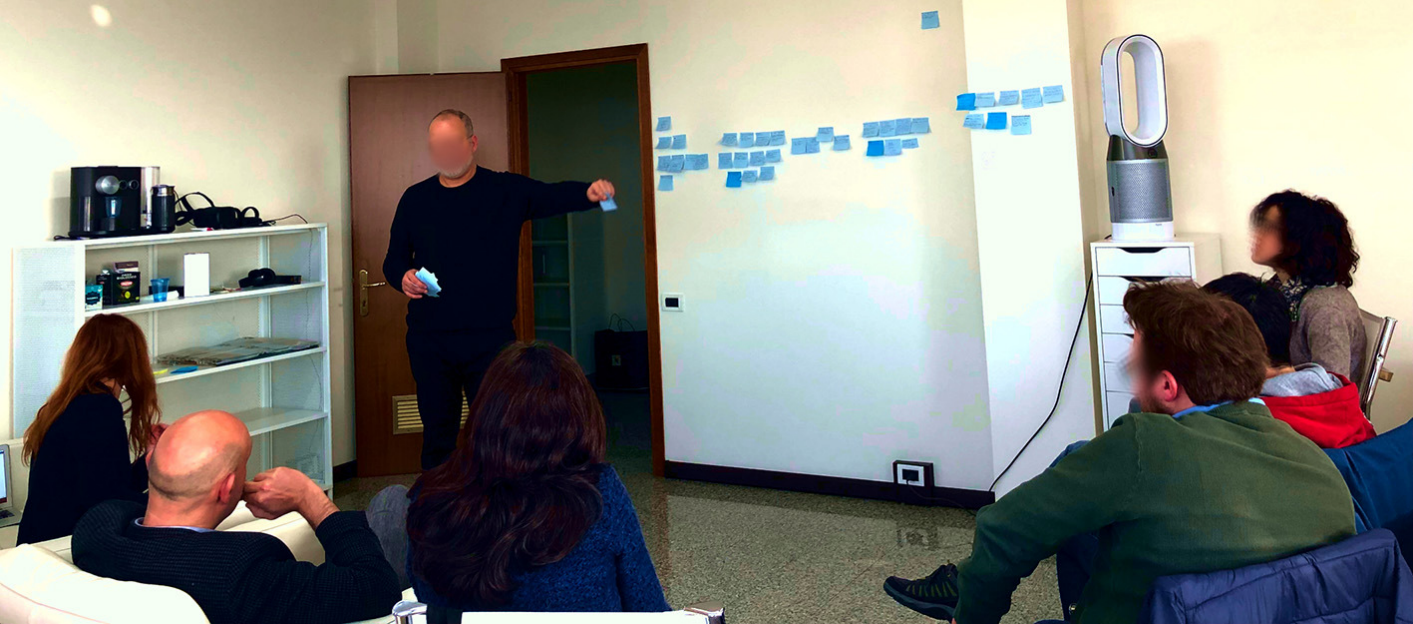
The first affinity diagram session.

The first session focuses on the time frame before the breakdown of the embankment and involved eight participants and one conductor. The sample included experts in hydrology (P1, P2, P3), hydraulics (P4, P5, P6), and psychologists with expertise in Human–computer interaction and new technologies (P8, P9). This heterogeneity made it possible to grasp every perspective related to the development of the scenario. The activity took place in a setting that favored the production and elicitation of ideas. The participants gathered in a room and arranged in a semicircle around the conductor. The conductor was a psychologist with prior experience in conducting affinity diagrams (P7). In a first phase, the conductor created a convivial atmosphere, introducing the activity, and the participants presented themselves and their expertise. Then, each participant had the task of answering the focus questions posed by the conductor relating to the situation prior to the collapse of the embankment. The questions were intentionally structured in a generic way, in order not to influence or limit the participants in producing the contents. More specifically they were: “What aspects of a flood emergency situation should be considered in a virtual simulation?” “How could these aspects be represented?” The conductor favored the emergence of spontaneous ideas about the topic analyzed, encouraging the use of creative and non-logical thinking. All participants produced a series of ideas and wrote every single idea on a different card (previously provided by the conductor) in the most concise and clear way possible. The ideas that emerged were subsequently read aloud by the conductor and discussed one by one among all the participants. The purpose of discussing ideas was to assign each of them to broader groups based on criteria similarity in order to organize what emerged into unanimously agreed groups (Figure 1). To do this, the generated cards were placed on a white wall, to help the participants in the process and to have an overall view. During this phase, the conductor encouraged people to contribute their points of view. The groups obtained were then unanimously labeled: the participants read the contents of each group and wrote the name that best represented each category on a new card. Furthermore, some categories were grouped into named macro-categories. Finally, the categories were hierarchized and related to each other. During the activity, the participants were involved and proactive, producing various ideas and actively participating in the discussion. The session lasted about 3 h.

#### Second Session: After the Embankment Breakdown

The sample of the second session consisted of seven participants (and one conductor), including expert in hydrology (P2), experts in hydraulics (P4, P5, P6), psychologists with expertise in Human-computer interaction (P8, P9), and a designer of virtual environments (VEs) (P10). The same conductor of the first activity, a psychologist with prior experience in conducting affinity diagrams (P7), supervised the session. The activity concerned the temporal window following the break of the embankment. The setting and the procedure employed were the same of the affinity diagram carried out in the first session. The same formulation of the key questions was adopted but focused on the post-break situation. The ideas that spontaneously emerged in response to the conductor's focus question were discussed, organized, and hierarchized, similarly to what was done previously. The conductor managed the entire session by moderating the discussion among the participants for a total duration of 3 h.

### Planning the Virtual Experience: Brainstorming Session

The results obtained from the activities were discussed and analyzed for the realization of the VR scenario proposals. To define the experience, a brainstorming session was done with one expert in Human–computer interaction (P8) and the two VE designers (P10, P11). Another expert in Human–Computer Interaction (P9) moderated the session. This co-design technique allows to involve participants in the generation of solutions in an informal and relatively unstructured way, with a judgment-free discussion (Dix et al., 2004). The available data from the two affinity diagrams were analyzed and translated into operational scenarios, considering the diversity of requirements that emerged. In particular, during a first phase most of the aspects highlighted during the previous activities were selected, giving particular importance to the contents that emerged as priorities in the hierarchy phase, and the ideas that emerged in both affinity diagrams. Then, the information was organized into a coherent narrative. Regarding the interaction with virtual space, different techniques and methods were considered by the same participants, taking into consideration previous studies, until a unanimous agreement was reached. The brainstorming lasted 4 h.

## Results

This section will illustrate the main results of the collaborative design sessions previously described.

### First Affinity Diagram Session: Risk Identification

**Figure 2:**
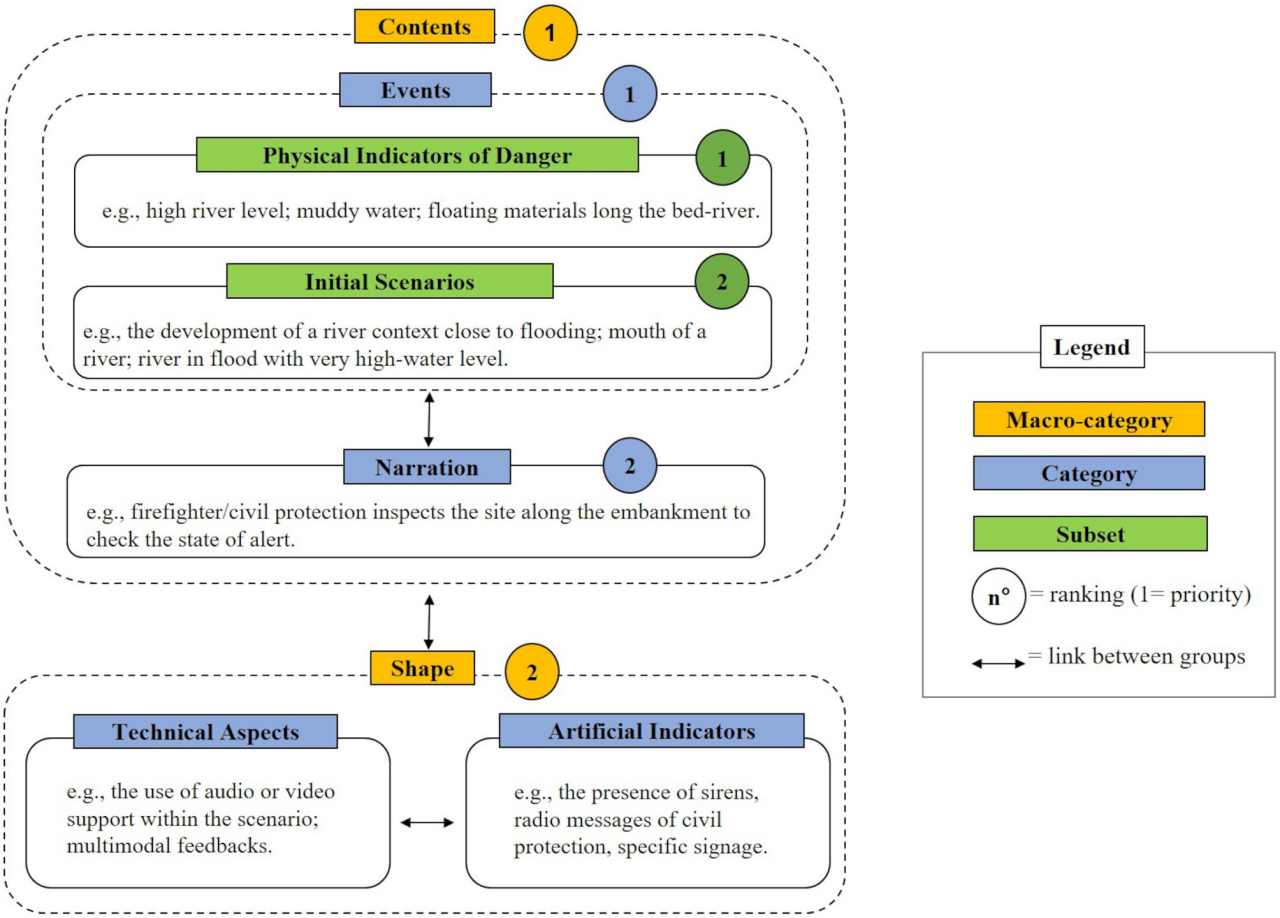
Schematic representation of the results of the first affinity diagram session.

Overall, in the first affinity diagram session related to the time before the embankment breakdown, several ideas were produced. The categorization and hierarchization of these ideas highlighted macro-categories, categories, and specific subsets (Figure 2). Two main macro-categories emerged, namely *Contents* and *Shape*, that users regarded as closely linked to each other for the experience.

The *Contents* macro-category was composed by the categories *Narration* and *Events*. In particular, *Narration* referred to the various narrative expedients that could be implemented to introduce the user to possible events (e.g., the possibility of bringing the user closer to the river in a scenario that includes a walk along the embankment; to be firefighter/civil protection that inspects the site along the embankment to check the state of alert). With *Events*, participants identified both the *Initial Scenarios* subset, that is the situations that may be represented in the VE (e.g., the development of a river context close to flooding, the mouth of a river, and a river in flood with very high-water level), and the subset *Physical indicators of danger*. The latter referred to natural signals that indicate an impending flood or possible subsidence of the embankment (e.g., the presence of flooded manhole covers, whirling eddies, floating material). These were the indicators that all the participants considered characterizing elements of the scenario for the educational objective.

The *Shape* macro-category referred instead to the formal aspects of the simulation. It was characterized by two categories, respectively, *Technical aspects* and *Artificial indicators*. The first one included the presentation modalities (e.g., the use of audio or video support within the scenario, multimodal feedbacks) and the possible methods that could be adopted to make the physical indicators of danger more evident (e.g., positioning a reference object in the river, such as a bridge, to show the height of the water level). Finally, in the *Artificial Indicators* category were collected the unnatural elements of alert (e.g., the presence of sirens, radio messages of civil protection, specific signage). Participants considered the two categories linked, because both provide elements to help the user identify the risk situation.

### Second Affinity Diagram Session: Facing the Emergency

**Figure 3:**
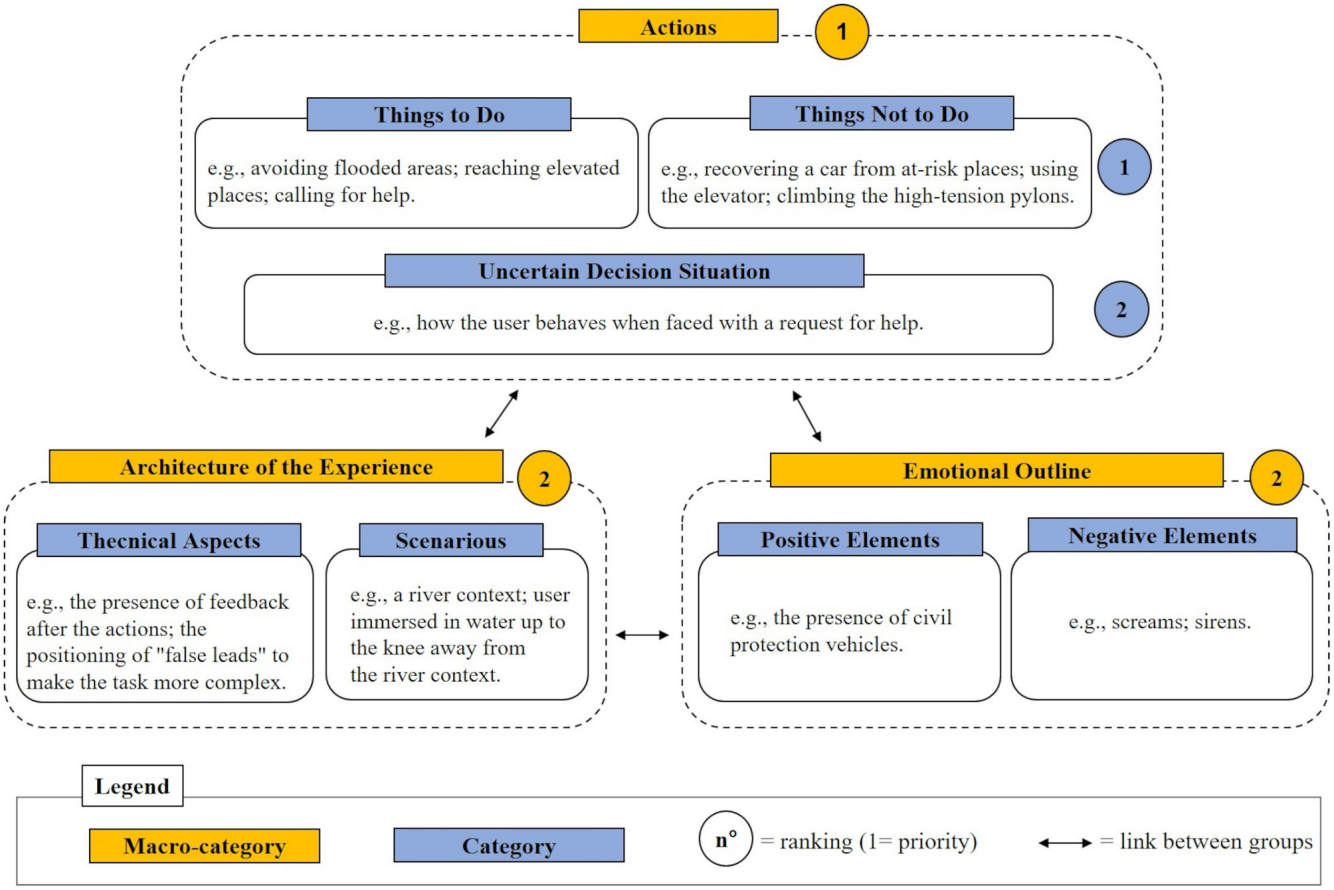
Schematic representation of the results of the second affinity diagram session.

From the second session, focused on the emergency following the breakdown of the embankment, three distinct macro-categories were collected, namely *Actions, Architecture of the experience*, and *Emotional outline*, with their specific categories (Figure 3). These groups were connected to each other by the users because they were closely related to the realization of the scenario.

The *Actions* macro-category includes the content aspects of the experience and focuses on the possible behaviors that could be taken during the emergency. These in turn have been categorized based on their correctness, with particular reference to the category *Things to do*, or rather the correct procedures to be adopted in the event of a break in the embankment, and *Things not to do*, or behaviors to avoid because they are considered dangerous or counterproductive during the emergency. The *Things to do* category includes, for example, actions such as avoiding flooded areas, reaching elevated places, calling for help. While, in *Things not to do*, various behaviors to avoid emerged, such as recovering a car from at-risk places, using the elevator, climbing the high-tension pylons. Besides, a third category named *Uncertain decision situations* referred to ambiguous situations that characterized an emergency (e.g., how the user behaves when faced with a request for help). This macro-category was identified by the participants as the most important of the three, with particular reference to “things to do” and “things not to do” categories.

The *Architecture of the Experience* macro-category included *Technical aspects* and *Scenarios*. In line with what emerged in the diagram relating to the pre-flood situation, *Technical aspects* refers to the presentation modalities of the contents (e.g., the presence of feedback after the actions, the positioning of “false leads” to make the task more complex). The *Scenarios* contains possible specific situations that could be represented (e.g., a river context, user immersed in water up to the knee in a non-river context).

Finally, macro-category named *Emotional outline* signaled the importance of creating situations with a high emotional impact and classifies the elements that could influence the user's emotional state with *Positive elements* (e.g., the presence of civil protection vehicles) or creating a greater alarm whit *Negative elements* (e.g., screams, sirens).

### Brainstorming Session: Finalization of the Virtual Simulation Design

Based on what emerged from the affinity diagrams, the brainstorming session led to the identification of two educational and engaging scenarios: the first one focused on the time frame that precedes the breakdown of the embankment (i.e., preparedness) and the second one focused on the post-breakdown emergency (i.e., response). Both scenarios have been designed to be usable both continuously, with chronologically ordered events, and separately, by having the breaking of the embankment as the final salient element of the experience (first scenario) or as the initial one (second scenario). The focus of the experience was on the *Physical indicators of danger* before the embankment's breakdown and on potential *Actions* to be performed or avoided during the consequent evacuation, because these were the contents that emerged as priorities in the hierarchy phase.

**Figure 4:**
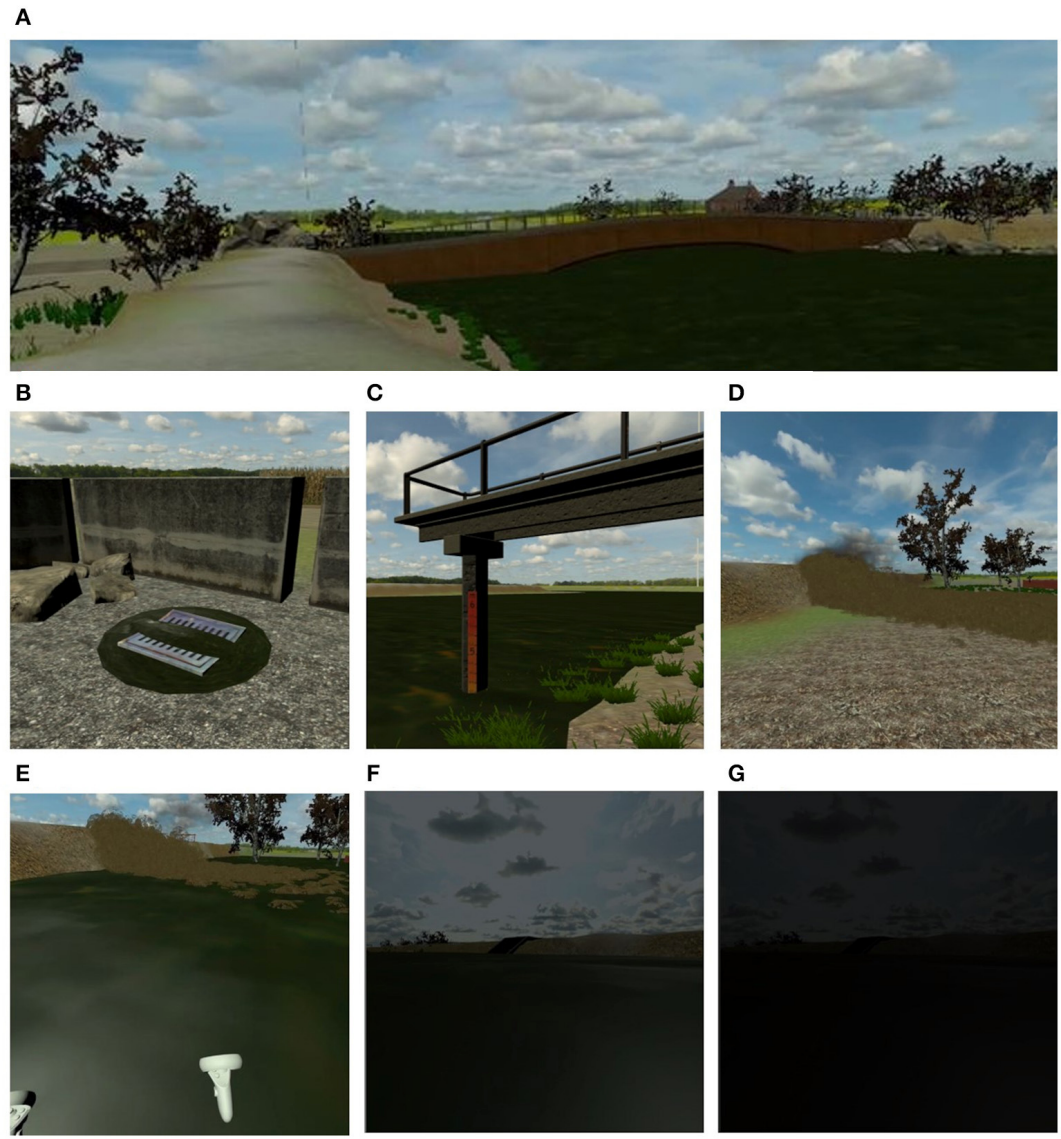
**(A)** A frame of the VE representing the initial part of the path along the river area. **(B)** A flooded manhole, one of the physical indicators of danger. **(C)** A hydrometer, an example of artificial indicator that facilitates the recognition of the high water level. (**D**) The breakdown of the river embankment. **(E)** While the water level rises, **(F)** the user fails to reach a high point, and **(G)** the screen slowly fades to black.

The VE depicts a river area and the neighboring landscape (Figure 4A). To ensure the realization of an ecological scenario and a realistic simulation, the environment is characterized by the typical sounds of nature, the specific fauna that populates the river, and the presence of other citizens (e.g., fishermen).

In the first scenario, the focus is on the exploration of risk indicators in order to increase user awareness. For this purpose, five *Physical indicators of danger* of an impending flood or a possible bank failure have been selected and inserted in the VE: (1) flooded manholes covers (Figure 4B), (2) high river level and muddy water, presence of (3) floating material, (4) whirling eddies, and (5) “fontanazzi” (leakage of cloudy water near the embankment). Moreover, the scenario presents a bridge (*Technical aspects*) and typical *Artificial indicators* of the river context (i.e., hydrometers positioned along the bridge and along the staircase) in order to make the “slow” physical indicators of danger (i.e., the water level) more easily recognizable (Figure 4C). The *Narrative* invites the user to explore the river environment and identify potential risk signs of levee breakage. To do this, the simulation begins with the user (who impersonates a member of the civil protection service) in a parking lot near a river. A colleague informs him/her via a phone call (i.e., an audio directly into the headset) that (s)he is there to inspect the state of the embankment, by checking if there are any indicators of danger (the actual task of the first scenario). The user then follows the path, walking along the embankment between the two sides of the river, and at the end of the task another phone call invites him/her to join the colleagues in a specific point of the VE. While the user approaches the indicated area, the narrative involves the subsidence of a point of the embankment (Figure 4D). The embankment breaks at a distance that does not physically overwhelm the user, but is sufficient to be able to observe the phenomenon, generating an immediate emergency. This causing the surrounding countryside to flood, with the water level rising (Figure 4E). The second scenario is immediately subsequent the breakdown of the embankment and focuses on learning the correct behaviors to adopt during an emergency. In this case, the narrative requires the user that is in a countryside bank to evacuate the area and to stay safe. To do this, as the water level rises, voices in the distance yell to the user to save themselves. The scenario offers to the user various escape routes and provides different feedbacks depending on the choices made. Based on what emerged in the affinity diagram relating to the post-breakdown situation, in the simulation are implemented elements that lead to safety, saving himself/herself in a high and protected location (*Things to do)*, but also stimuli that could lead to dangerous conduct (e.g., climb a tree that is not resistant enough or take a car that is in at-risk place). The actions performed by the user will be followed by adequate immediate feedback according to the correct or incorrect behavior, from an educational point of view. Once a stable and safe position has been reached, the user must contact the emergency services (and not clog the lines by calling friends or family). Another aspect considered in the scenario is related to the *Emotional outline*, with the insertion of citizens running away (*Negative aspects*). Finally, in both scenarios, elements of gamification (*Technical aspect*) were considered, such as the possibility of assigning scores to the user based on his/her actions or of running into a “game over” condition in the event of critical errors. These elements, typical of serious games, help in making the experience not only educational but also challenging. Also, they lighten the tone of the intrinsically dramatic situation represented and could be experienced as excessively stressful by users. The simulation ends when the user succeeds in calling emergency services.

### Implementation of the Virtual Simulation

The two scenarios defined during the brainstorming activity were created using Unity (version 2020.1.17). In particular, the 3D models were built with blender 2.9. To make the simulation more realistic, the VE was partially based on a river area in northern Italy known to be subject to frequent floods. The ambient audio used was recorded with a binaural microphone (Neumann KU100) in the field (i.e., river area). The resulting simulation has been configured to be usable through Oculus Quest 2 (resolution: 1,832 × 1,920 px per eye; refresh rate: 90 Hz; Field of view: 100-degree est.; integrated speakers and microphone).

To select elements in the VE (e.g., an indicator of danger) the “ray casting” method (using the trigger button of the Quest controllers) was employed. As locomotion modality, “teleportation” was selected (assigned to the thumb stick button). Both the chosen techniques are widely used in the field of VR and well-received by users, in terms of ease of use, cognitive load, and cybersickness (Nukarinen et al., 2018; Loup and Loup-Escande, 2019). Finally, on the basis of what emerged in the brainstorming session, an immediate feedback system was adopted in the simulation, similarly to Chittaro and Buttussi (2015). In case of adoption of an incorrect behavior or omission of a correct one, a negative feedback (with visual, vibrotactile, and auditory elements) is provided, followed by a short recommendation. For example, if the user fails to reach a high point during the flooding, the sound of the water intensifies, the controller vibrates, the screen fades to black (Figures 4F,G), and then the user receives the message “Reach a location at a safe altitude.” Then, the user starts over from the point in the narrative where the mistake was made.

The simulation collects and logs the number of physical indicators of danger found, their type, when they were identified, the data relating to the actions performed, the path taken by the users (as a sequence of spatial coordinates), and the overall time spent in the scenario by the user.

## Discussion

The purpose of the present study was to describe the co-design activities implemented to identify the contents of the VR simulation “Safer Water” for river flood emergencies. The focus was on designing virtual scenarios that would provide useful knowledge for users to deal with an embarkment breakdown with consequent flooding, with the ultimate aim of improving the quality of life of those who live near a river. To this end, experts with different theoretical backgrounds were involved in two affinity diagram sessions and a brainstorming session.

Overall, the co-design process we adopted for the creation of the VE has led to a series of advantages. Previous studies have shown how co-design techniques can be effectively used to design virtual scenarios related to specific situations, as it allows to obtain valuable information from experts in the reference sector (Eisapour et al., 2020). In our case, we have chosen to also involve HCI experts and VE designers from the beginning, a choice that was made only in a few of the previous works (Bettelli et al., 2019; Tabbaa et al., 2020). Involving this type of experts from the early design stages allowed us to focus not only on the contents of the VR environment (e.g., danger indicators of a flood, correct evacuation behaviors), but also on the technical aspects and interactive modalities (e.g., feedback, gamification elements), obtaining a global view of the various parts to be taken into consideration. We also found useful having carried out more than one affinity diagram session since it let us analyze the two different phases of the emergency separately. Indeed, emergency situations are complex phenomena, and having the possibility to break them into multiple phases with related activities was an aspect that facilitated the co-design process, giving the participants the time necessary to produce ideas and investigate in detail a series of elements related to a specific time frame of the natural disaster. However, it should be considered that if on the one hand doing multiple sessions allows a deeper analysis of the phenomenon, on the other it means risking not having the same participants in all the sessions.

Regarding the results of the co-design activities, two related virtual scenarios with different focuses emerged: a first scenario aimed at identifying the signs of a probable breakdown of the embankment (i.e., preparedness), and a second one concerned the behaviors to be adopted during the emergency (i.e., response).

Different narrative modes were associated with the different objectives. In the first scenario, an exploratory modality, based on researching and detecting the cues, was selected. In the scenario related to the situation following the embankment breakdown, an interactive-experiential modality was chosen: the user receives positive or negative feedback based on the correctness of his/her behavior in the VE.

Compared to previous literature on VR and river flood emergency, our results highlighted three fundamental aspects. First, the need to consider not only the emergency in progress but also the time frame before the disaster has clearly emerged. Instructing users on the environmental elements to pay attention to in order to estimate the probability of an embankment breakdown could be fundamental to increase their risk awareness and well-being. Indeed, such calamities are often perceived as sudden and unpredictable events, and we expect that providing citizens with the knowledge to grasp the signs that precede them can improve their perception of control over the environment and, consequently, their quality of life. However, previous works on the topic largely ignored this aspect by placing the user already in the emergency or shortly before its occurrence (Zaalberg and Midden, 2013; Fujimi and Fujimura, 2020).

Secondly, from the affinity diagram and the brainstorming sessions, a preference to present the indicators of danger in a natural and realistic way emerged in order to facilitate the transfer of the information learned into a real context. For example, the river's water level in our virtual scenario is already relatively high, an aspect that can be identified thanks to references such as bridges or hydrometers along the path. Previous works have instead preferred to artificially accelerate the time flow in the simulation, a system that allows researchers to show in a few minutes changes that occur over hours at the cost of diminishing the realism of the experience (Fujimi and Fujimura, 2020; Skinner, 2020).

Finally, the co-design activities made it possible to highlight which actions could be done and which should be avoided during the flood emergency, giving immediate feedback to the user. The possibility of letting users experience firsthand the consequences of their behavior in an immediate and vivid way was a design choice adopted in various studies on emergency and risk situations (Chittaro and Buttussi, 2015; Buttussi and Chittaro, 2018; Feng et al., 2018; Van Ginkel et al., 2020), but still little used in the works concerning specifically the flood emergency, more centered on allowing the users to visualize the disaster and not to actively act to face it. In particular, a preference for an immediate feedback system emerged from the co-design activities. The literature on the subject is not conclusive: in the context of VR emergency training, both immediate and delayed feedback have been argued to be effective to enhance trainees' preparedness (Feng et al., 2021). Some evidence in favor of deferred feedback was found with child users (Feng et al., 2021), while in the case of adults the question is still open. However, besides the timing, the way in which feedback is provided is also of considerable importance. In our case, it was designed not only to suggest the appropriateness of a behavioral choice but also as a tool to increase the emotional arousal. For example, if the user performs an incorrect action, (s)he does not just read a canvas that signals the error but experiences the negative consequences firsthand. As reported by previous work in other emergency context, the inclusion of emotionally intense consequences of typical mistakes could promote knowledge retention (Chittaro and Buttussi, 2015) and therefore represents an example of a psychologically appropriate way to provide information.

To summarize, the immersive and interactive “Safer Water” experience was designed with the aim of increasing awareness on how to best manage an alert or emergency situation, with the final goal of improving the quality of life of citizens who live close to at-risk rivers. This simulation could be used within training or education courses in different contexts, such as school, business, or within thematic meetings on flood problems organized by local authorities, such as the municipality or civil protection, for raising awareness in the community. In other contexts, the integration of similar VR experiences has already shown to help users in staying engaged and keeping their attention focused (Sacks et al., 2013; Chittaro and Buttussi, 2015).

In the following subsections, the salient points that emerged during the co-design activities and on which the “Safer Water” simulation was based, are reported in the form of guidelines. Although many of the guidelines are related to the specific situation of breakdown of the embankment, others may be generalizable to other emergency contexts.

### Guidelines for Virtual Simulation Design of Flood Emergency

This research provides information relating to the design of virtual simulation about flood emergency. The guidelines proposed below are the result of the salient findings that emerged from the co-design sessions. All the guidelines were organized into groups. In the first one, general information regarding the VE design related to flood emergency (scenarios and system design) is reported. The second group presents natural risk indicators of a flood emergency related to the possible breaking of an embankment (physical indicators of danger). Finally, the third one refers to which actions should be taken and which should be avoided during the flood emergency (facing the emergency).

#### Scenarios and System Design

- The simulation should reproduce the salient event relating to the breakdown of the embankment.
- The scenario should include situations that allow the user to face the emergency by actively experiencing the effects of his/her behavior.
- The scenario should provide clear and immediate feedback on the correct and incorrect choices made by users.
- The scenario should include not only emergency management situations, but also risk identification.
- The scenario should include salient indicators of danger.
- In the simulation, the indicators of danger should be represented with realism and their timing.
- The simulation should include emotional stimuli to make the experience realistic.
- The simulation should include social stimuli that recall an ecological situation.
- Gamification elements should be included in the simulation to involve the user in the educational experience and not generate a situation of excessive stress for the user.
- The architecture of the scenario should be modular for overall or partial use of the simulation, according to the different objectives and contexts.
- The scenario should include environmental sounds related to flora and fauna of the river area to make the VE more realistic.
- The scenario should include the implementation of spatialized audio to increase the level of immersion.
- The scenario should include facilitators (e.g., bridges, hydrometers) to help users identify physical indicators of danger.
- Natural barriers should be inserted in the environment to limit ecologically the space that can be explored by the user.

#### Physical Indicators of Danger

- Presence of rising water from the subsoil (e.g., flooded manhole, puddles).
- Presence of high river level.
- Presence of muddy water.
- Presence of floating materials long the bed-river (e.g., branches, waste).
- Presence of whirling eddies along the bed-river, with particular reference to the riversides.
- Presence of leakage of cloudy water near the embankment (“fontanazzi”).

#### Facing the Emergency

- If possible, prefer paved areas over the ground.
- In an outdoor situation, reach sites at a safe altitude and avoid areas of depression (e.g., avoid underpasses).
- In an indoor situation, go to the upper floors and avoid basements.
- Inside buildings, disconnect the power.
- Inside buildings, avoid using elevators.
- Avoid contact with electrical sources.
- Reach any designated collection points in case of municipal civil protection plans.
- Avoid saving movable objects from the lower floors to the upper floors (only basic necessities).
- Avoid crossing bridges when the water level is very high.
- Use the telephone lines only to contact emergency services.
- Do not drive in flooded areas.
- Do not drink tap water during an emergency situation.

## Limitations and Future Works

Some limitations of the present work should be acknowledged. The first one includes not having adequately considered how to manage flood emergency situations in indoor contexts. In fact, although in the affinity diagram sessions elements relating to the behaviors to be kept within domestic environments emerged, it was preferred in the resulting scenarios to focus on the outdoor context. Future studies will have to investigate this aspect, designing virtual simulations that help citizens adopt safe behaviors during a flood emergency, even inside their homes. Second, not all the participants were able to take part in both the first and the second affinity diagram sessions. Although there were an adequate number of experts on river flooding and new technologies in both activities, the lack of some participants may have influenced the ideas that emerged. Finally, as mentioned earlier in the discussion, some of the guidelines proposed (e.g., those in subsection Physical indicators of danger) concern specifically the river flood emergency and are difficult to generalize to other situations.

The co-design activities previously described have relied on expert users. The next steps will consist in involving citizens who live in areas at risk or people who have experienced flood emergencies in the past to understand their needs and concerns to improve the virtual experience. A series of evaluation will be carried out to test the effectiveness of the design choices adopted, to verify how the simulation is received by the end users, and to validate the guidelines emerged from the activities.

In our case, the co-design methodology was applied to a very specific situation, but future research should expand its application to the design of VR simulations of other emergency situations, an area not adequately covered at the moment.

## Conclusions

The present work describes how co-design methodologies can be used to identify the contents of a VR simulation for river flood emergencies. Two affinity diagram sessions and a brainstorming were conducted with experts with different theoretical backgrounds, while keeping in mind that the ultimate goal was to improve the quality of life of citizens exposed to risk of floods. From the activities, a series of results emerged. First, the adopted method made it possible to design a VR application that focused on two main aspects of a flood situation: the identification of the signs of a probable breakdown of the embankment and the adoption of the correct behaviors during the emergency. Second, the key points that emerged allowed us to highlight a set of guidelines to support the design of VR simulation for the purposes discussed in the paper.

## Data Availability Statement

The raw data supporting the conclusions of this article will be made available by the authors, without undue reservation.

## Ethics Statement

Ethical review and approval was not required for the study on human participants in accordance with the local legislation and institutional requirements. The patients/participants provided their written informed consent to participate in this study.

## Author Contributions

LG and MF conceived and planned the work and the methodological approach. LG coordinated the project development and moderated the affinity diagram sessions. GB moderated the brainstorming session. AB and GB took part as HCI experts to the affinity diagram sessions and analyzed the data. AB, VO, AS, and LG wrote the manuscript. All authors contributed to the article and approved the submitted version.

## Conflict of Interest

The authors declare that the research was conducted in the absence of any commercial or financial relationships that could be construed as a potential conflict of interest.

## Footnotes

^1^*WHO. Floods*. https://www.who.int/health-topics/floods#tab=tab_1).
